# Estimating Tea Plant Physiological Parameters Using Unmanned Aerial Vehicle Imagery and Machine Learning Algorithms

**DOI:** 10.3390/s25071966

**Published:** 2025-03-21

**Authors:** Zhong-Han Zhuang, Hui-Ping Tsai, Chung-I Chen

**Affiliations:** 1 Department of Civil Engineering, National Chung Hsing University, Taichung 402, Taiwan; ken870315@smail.nchu.edu.tw; 2 Innovation and Development Center of Sustainable Agriculture, National Chung Hsing University, Taichung 402, Taiwan; 3 i-Center for Advanced Science and Technology (i-CAST), National Chung Hsing University, Taichung 402, Taiwan; 4 Smart Multidisciplinary Agriculture Research and Technology Center, National Chung Hsing University, Taichung 402, Taiwan; 5 Department of Forestry, National Pingtung University of Science and Technology, Pingtung 912, Taiwan; cichen@mail.npust.edu.tw

**Keywords:** unmanned aerial vehicles, tea plants, machine learning, leaf area index, photochemical reflectance index, quantum yield of photosystem II

## Abstract

Tea (*Camellia sinensis* L.) holds agricultural economic value and forestry carbon sequestration potential, with Taiwan’s annual tea production exceeding TWD 7 billion. However, climate change-induced stressors threaten tea plant growth, photosynthesis, yield, and quality, necessitating an accurate real-time monitoring system to enhance plantation management and production stability. This study surveys tea plantations at low, mid-, and high elevations in Nantou County, central Taiwan, collecting data from 21 fields using conventional farming methods (CFMs), which emphasize intensive management, and agroecological farming methods (AFMs), which prioritize environmental sustainability. This study integrates leaf area index (LAI), photochemical reflectance index (PRI), and quantum yield of photosystem II (ΦPSII) data with unmanned aerial vehicles (UAV)-derived visible-light and multispectral imagery to compute color indices (CIs) and multispectral indices (MIs). Using feature ranking methods, an optimized dataset was developed, and the predictive performance of eight regression algorithms was assessed for estimating tea plant physiological parameters. The results indicate that LAI was generally lower in AFMs, suggesting reduced leaf growth density and potential yield differences. However, PRI and ΦPSII values revealed greater environmental adaptability and potential long-term ecological benefits in AFMs compared to CFMs. Among regression models, MIs provided greater stability for tea plant physiological parameters, whereas feature ranking methods had minimal impact on accuracy. XGBoost outperformed all models in predicting parameters, achieving optimal results for (1) LAI: R^2^ = 0.716, RMSE = 1.01, MAE = 0.683, (2) PRI: R^2^ = 0.643, RMSE = 0.013, MAE = 0.009, and (3) ΦPSII: R^2^ = 0.920, RMSE = 0.048, MAE = 0.013. Overall, we highlight the effectiveness of integrating gradient boosting models with multispectral data to capture tea plant physiological characteristics. This study develops generalizable predictive models for tea plant physiological parameter estimation and advances non-contact crop physiological monitoring for tea plantation management, providing a scientific foundation for precision agriculture applications.

## 1. Introduction

Tea plants (*Camellia sinensis* L.) hold economic value in agriculture and possess the characteristics of an evergreen perennial in forestry. Beyond their potential for atmospheric CO_2_ sequestration [1,2,3], Tea-based beverages are rich in bioactive compounds, particularly polyphenols, which provide notable health benefits [4,5]. In Taiwan, annual tea production reaches 12,000 metric tons, with a total market value exceeding TWD 7 billion, highlighting the tea ecosystem’s sustainability and its crucial role in the agricultural economy. According to projections from the Taiwan Climate Change Projection Information and Adaptation Knowledge Platform (TCCIP), based on Intergovernmental Panel on Climate Change (IPCC) AR6 scenarios, Taiwan is expected to experience greater seasonal rainfall variability, along with an increased risk of spring droughts. Changes in precipitation and temperature will likely impact yield and distribution or raise the risk of wildfires [6,7,8]. The growing frequency of extreme weather events and water shortages will further stress photosynthetic efficiency in tea plants [9] and increase growth risks [10], ultimately affecting tea leaf quality [11]. Tea plants are predominantly cultivated in tropical, subtropical, and temperate regions at elevations below 3000 m [12]. Their bud growth rate is regulated by a combination of environmental factors and genetic traits [13]. Consequently, enhancing tea plant resilience to climate stress requires accurate, real-time physiological monitoring to enable early intervention strategies.

Photosynthesis drives the terrestrial carbon cycle and serves as a key indicator of crop growth, productivity [14], and adaptation to environmental changes. This study focuses on three physiological indices of tea plants: (1) leaf area index (LAI), (2) photochemical reflectance index (PRI), and (3) quantum yield of photosystem II (ΦPSII). LAI quantifies total leaf area, offering insights into canopy density [15], crop yield, and production efficiency [16], while also serving as a key regulator of photosynthesis, respiration, and rainfall interception [17,18,19]. PRI is closely related to photosynthetic efficiency [20] and a plant’s response to environmental stress [21]. PRI primarily reflects energy partitioning in the light-harvesting process, with typical values ranging from −0.3 to 0.3, where higher values indicate greater light use efficiency, while lower values suggest activation of photoprotective mechanisms. ΦPSII is a key indicator of photochemical efficiency, commonly used to assess photosynthetic performance and quantify the effects of environmental stress on light reactions [22]. ΦPSII values typically range from 0 to 0.85, with higher values indicating greater PSII activity, while values below 0.2 suggest that photosynthesis is inhibited.

Traditional crop monitoring relies on manual field inspections and point-based instrument sampling, which offer high accuracy and direct insights into vegetation structure, physiological status, and photosynthetic efficiency in situ. However, these methods are time-consuming and spatially limited [23]. As research demands increase, remote sensing technologies offer advantages for large-scale agricultural monitoring, encouraging a shift toward integrating ground-based measurements with remote sensing approaches. By using high-precision field measurements as reference values and leveraging the spatial coverage of remote sensing imagery, researchers can overcome the limitations of traditional methods [24]. Among these technologies, unmanned aerial vehicles (UAVs) provide rapid, non-destructive, and large-scale crop monitoring capabilities, offering essential spectral, high-spatial, and temporal resolution data for precision agriculture [25,26,27,28]. UAVs can be equipped with visible, multispectral, hyperspectral, and thermal sensors, capturing spectral imagery to extract key crop characteristics for monitoring field conditions [29,30].

For LAI estimation, previous studies have combined spectral information, texture features, and canopy structure [31,32,33]. Gong et al. [34] successfully developed a rice LAI estimation model using destructive sampling, UAV-derived visible imagery, and canopy height data, achieving a root mean square error (RMSE) below 1.1. Similarly, Ochiai et al. [35] used visible and multispectral imagery to estimate sweet potato LAI, showing that removing background noise and selecting optimal image features improved estimation accuracy, achieving an R^2^ of 0.887 using partial least squares regression. For PRI estimation, challenges arise due to the mismatch between the narrowband reflectance wavelengths required for PRI calculation and the available spectral bands of multispectral sensors [36]. However, previous research has demonstrated that field-measured PRI values are significantly correlated with chlorophyll content, carotenoid content, and the carotenoid-to-chlorophyll ratio [37]. As a result, alternative chlorophyll-sensitive indices have been proposed, such as the green chlorophyll index (GCI), chlorophyll vegetation index (CVI), and green normalized difference vegetation index (GNDVI), which exhibit moderate correlations with chlorophyll and carotenoid content [38]. Notably, GNDVI has shown greater sensitivity to chlorophyll concentration compared to the commonly used normalized difference vegetation index (NDVI) [39]. Estimating ΦPSII generally relies on hyperspectral sensors capable of capturing sun-induced chlorophyll fluorescence (SIF) signals, which are weak fluorescence emissions from photosynthetic pigments [40]. These emissions span the 650–800 nm range, with fluorescence peaks at 685 nm and 740 nm [41]. Sims and Gamon [42] further noted that visible wavelengths are primarily influenced by leaf surface structure, whereas near-infrared reflectance provides better insights into leaf internal structure and physical properties.

Despite the significant potential of UAVs in agricultural management and crop parameter estimation, challenges remain in applying spectral techniques for tea plant monitoring. Therefore, this study focuses on tea plantations at low, mid-, and high elevations in Nantou County, central Taiwan, collecting in situ LAI, PRI, and ΦPSII measurements from tea fields managed under the conventional farming method (CFM) and the agroecological farming method (AFM). Visible and multispectral UAV imagery was also acquired to develop a dataset of image-derived indices for constructing tea plant physiological parameter prediction models. The specific objectives of this study are (1) to investigate the effects of elevation and farming methods on tea plant physiological status through long-term in situ measurements; (2) to optimize feature selection by ranking image indices based on their relationships with tea plant physiological parameters; and (3) to evaluate the accuracy of eight regression models in predicting tea plant physiological parameters and further analyze model performance under different season, elevation, and farming method conditions, validating their applicability across diverse environmental conditions.

## 2. Materials and Methods

### 2.1. Overview

**Figure 1:**
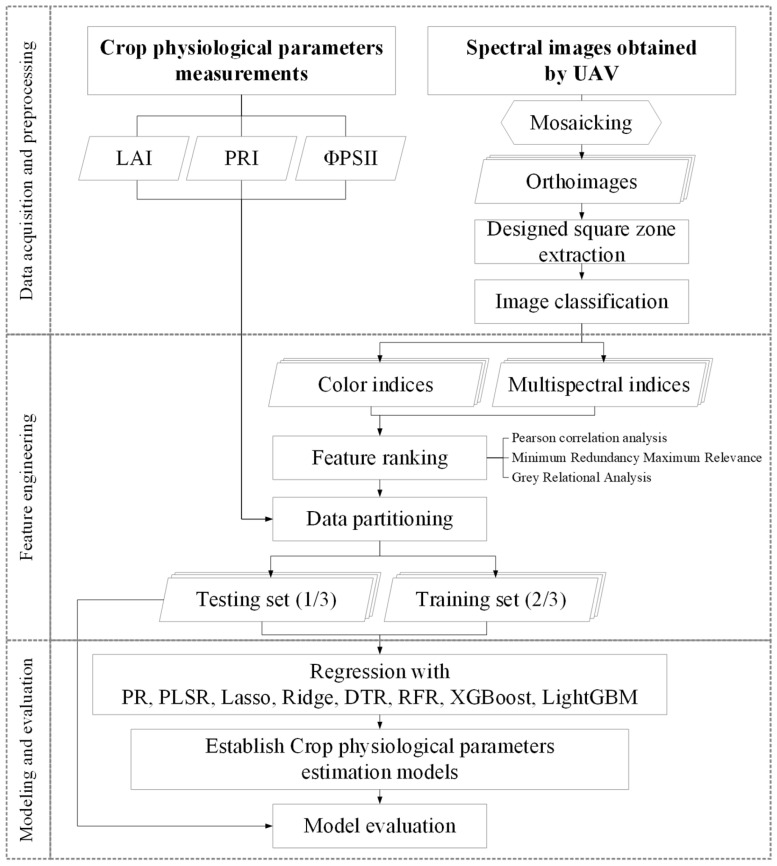
Research flow.

The overall workflow of this study is illustrated in Figure 1. It consists of three main steps: (1) data acquisition and preprocessing, (2) feature engineering, and (3) modeling and evaluation. In the data acquisition and preprocessing stage, physiological parameters, including leaf area index (LAI), photochemical reflectance index (PRI), and quantum yield of photosystem II (ΦPSII), were collected in the fields, while UAV flight missions were conducted to capture aerial imagery. The acquired images underwent post-processing, which included orthomosaic generation, image cropping, extraction of tea tree regions, and computation of image-based indices, including color indices (CIs) and multispectral indices (MIs). In the feature engineering stage, important features were ranked, and the dataset was divided into training and testing subsets. Finally, in the modeling and evaluation step, eight statistical and machine learning algorithms were applied to develop predictive models, with hyperparameter optimization performed to enhance model performance. The accuracy of the models was assessed, leading to the construction of predictive models for estimating tea tree physiological parameters.

### 2.2. Experimental Site and Design

**Figure 2:**
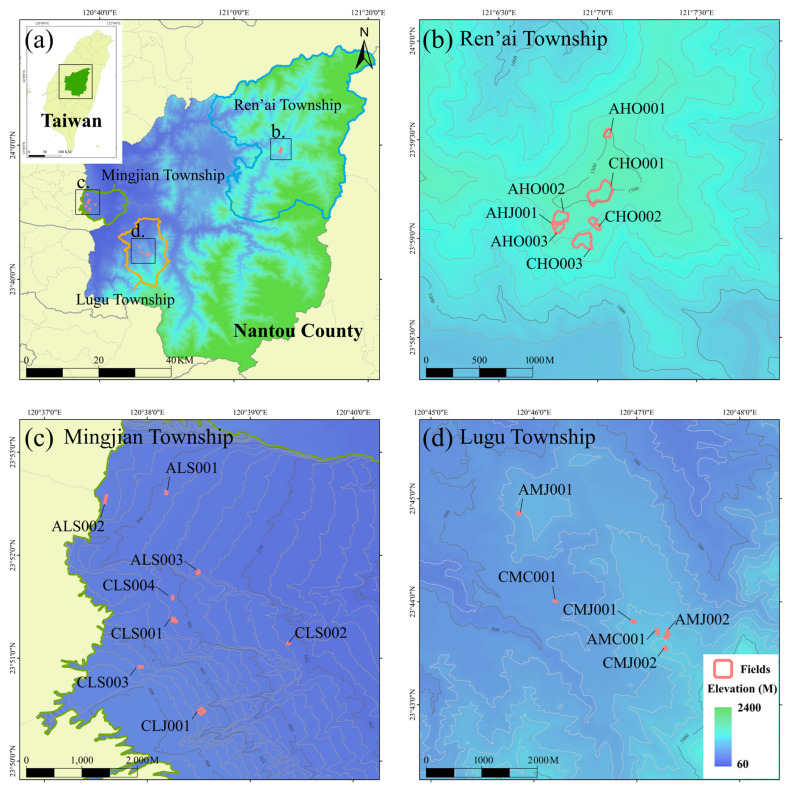
Geographical distribution map of the study plantations. (**a**) Geographic location and elevation of Nantou County. (**b**) Ren’ai Township. (**c**) Mingjian Township. (**d**) Lugu Township.

**Figure A1:**
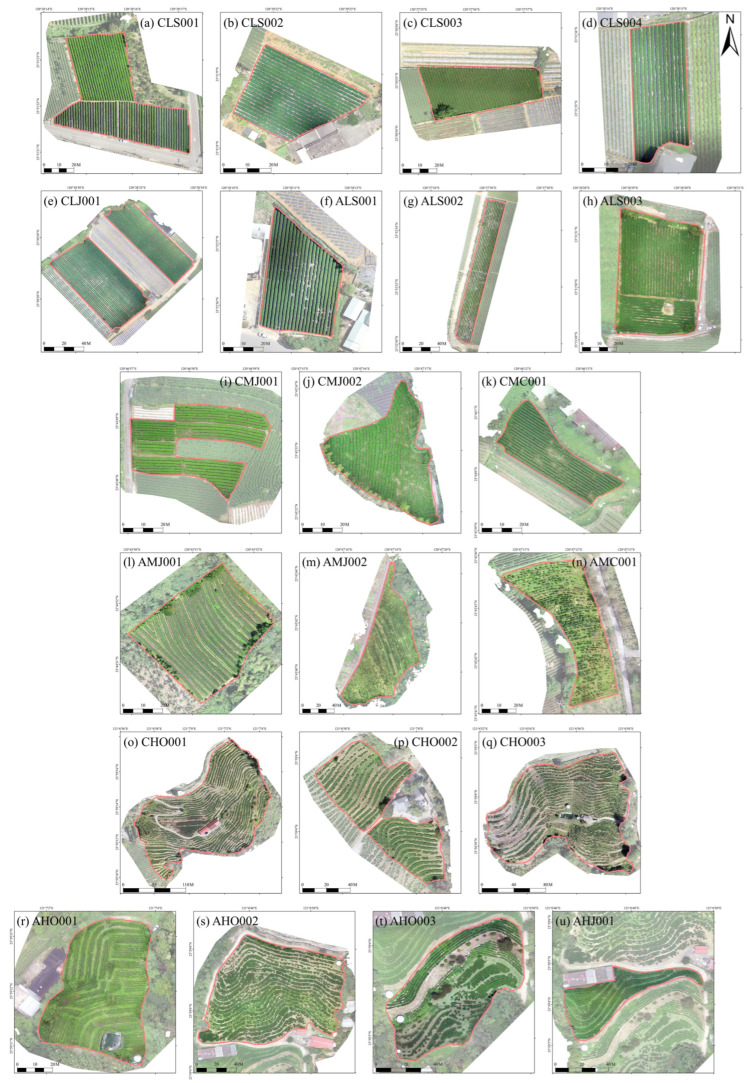
Orthomosaic images for each tea plantation.

The experimental tea plantations are located in Nantou County, central Taiwan, a key tea-growing region that accounts for approximately 60% of Taiwan’s total tea production, exceeding 7400 metric tons annually. To ensure the generalizability of this study, tea plantations at three different elevations were selected, encompassing both conventional farming methods (CFMs) and agroecological farming methods (AFMs). Regarding farming practices, the CFM involves more frequent human intervention, typically including regular weed removal, fertilization, and irrigation to ensure that tea plants receive a stable and sufficient supply of nutrients. In contrast, AFM emphasizes sustainable agricultural practices that promote ecological conservation, including reducing chemical fertilizer usage and enhancing environmental sustainability. The field survey was conducted from July 2021 to August 2024, with each experimental site surveyed once per month. Data collection was carried out sequentially based on elevation, meaning that all tea plantations within a given elevation were surveyed for one full year before transitioning to the next, ensuring that complete annual observational data were obtained for all sites at each elevation. This study focused on three elevation ranges (Figure 2). At low elevations (0~500 m) in Mingjian Township (120°37′34″ E~120°39′24″ E, 23°50′25″ N~23°52′39″ N), the predominant cultivar is Sijichun, with eight tea plantations selected for this study. At mid-elevations (500~1000 m) in Lugu Township (120°45′49″ E~120°47′20″ E, 23°43′32″ N~23°44′53″ N), the study area includes six tea plantations growing Chin-Shin-Dapan and Jinxuan. At high elevations (1000~1500 m) in Ren’ai Township (121°4′46″ E~121°7′5″ E, 23°58′56″ N~23°59′34″ N), seven tea plantations were selected, primarily cultivating Chin-Shin-Oolong. Additionally, Jinxuan, known for its strong environmental adaptability, was included in 1 to 2 plantations at each elevation. The climate of the study area is classified as subtropical to temperate, with an annual mean temperature ranging from 18 to 24 °C and an average annual precipitation of 1700 to 2600 mm. The orthomosaic images of each tea plantation are provided in Figure A1, while the basic information for each field is shown in Table A1.

### 2.3. Data Acquisition

**Figure 3:**
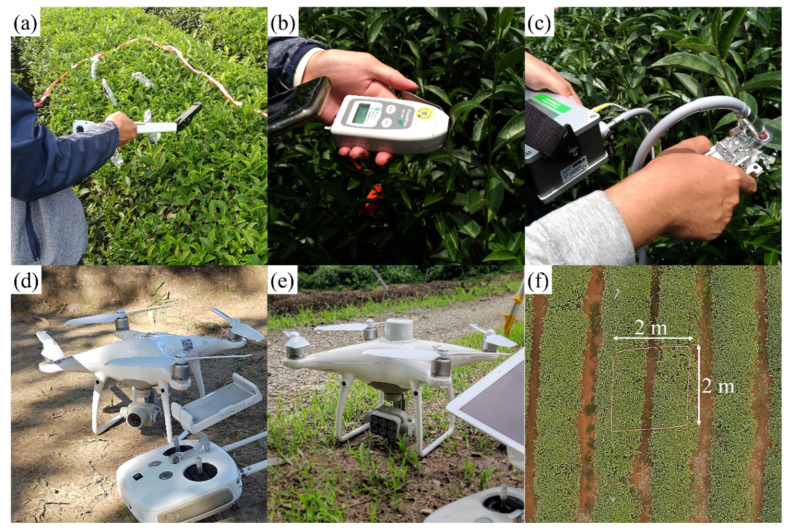
Field measurement instruments and designed square zone (DSZ). (**a**) LAI-2200C Plant Canopy Analyzer. (**b**) PlantPen PRI 200. (**c**) MINI-PAM-II photosynthesis yield analyzer. (**d**) DJI Phantom 4 Pro. (**e**) DJI Phantom 4 Multispectral. (**f**) Designed square zone.

Field data collection was divided into two main components: (1) physiological measurements of tea plants and (2) UAV imagery acquisition. The physiological measurements included the leaf area index (LAI) (Figure 3a), photochemical reflectance index (PRI) (Figure 3b), and chlorophyll fluorescence parameters (Figure 3c), while UAV imagery was collected using visible-light (Figure 3d) and multispectral sensors (Figure 3e). To ensure data consistency and spatial correspondence, physiological parameters and images were collected on the same day. In each sample site, three to five rectangular experimental zones with dimensions of 2 m in length and width were established, referred to as designed square zones (DSZs), which served as corresponding areas for physiological data measurements and image analysis (Figure 3f).

#### 2.3.1. Tea Plant Physiological Parameter Acquisition

The leaf area index (LAI) is defined as the total leaf area per unit of projected ground area [43]. It reflects the density of crop leaf area and serves as a crucial parameter for assessing canopy coverage, growth, and yield potential [44]. In this study, LAI measurements for tea plants were conducted using the LAI-2200C Plant Canopy Analyzer (Li-Cor, Inc., Lincoln, NE, USA). The measurement process involved analyzing incident light from five different fields of view (FOVs). First, a reference measurement was taken in an open area to establish baseline light intensity, followed by a measurement beneath the crop canopy to assess transmitted light. The LAI value for each DSZ was computed using software algorithms.

The photochemical reflectance index (PRI) is a remote sensing method for assessing photosynthetic efficiency based on reflectance measurements [45,46]. In this study, PRI was measured using the PlantPen PRI 200 (Photon Systems Instruments Ltd., Brno, Czech Republic). Leaf clips were used to record spectral reflectance values from tea leaves, with 10 measurements taken per DSZ, and the average value was used as the PRI for that DSZ. The index is calculated from the reflectance at 531 nm and 570 nm using the following formula:

(1) $$PRI=\frac{R_{531}-R_{570}}{R_{531}+R_{570}}$$

$R_{531}$ and $R_{570}$ represent the green reflectance at 531 nm and the yellow reflectance at 570 nm, respectively [47]. The green band is closely related to the de-epoxidation state of the xanthophyll cycle, which plays a key role in regulating excess light energy dissipation [48,49]. Additionally, long-term variations in PRI values are influenced by changes in the size of constitutive pigment pools within leaves [50], reflecting the plant’s regulatory mechanisms for long-term environmental adaptation. Under environmental stress conditions, plants activate the xanthophyll cycle to dissipate excess light energy, thereby reducing photoinhibition damage [47]. Furthermore, PRI has been used to monitor crop water stress [51,52] and to assess photosynthetic efficiency at both leaf and canopy scales [53].

This study monitored chlorophyll fluorescence parameters and calculated the quantum yield of PSII (ΦPSII), which is an important parameter reflecting the photochemical processes in plants. By measuring the linear electron transport rate, ΦPSII describes the proportion of absorbed light energy utilized by photosystem II (PSII) for photochemical reactions [54,55]. The MINI-PAM-II photosynthesis yield analyzer (MINI-PAM-II, Walz, Germany) was used for measurements, with ΦPSII calculated using the following formula:

(2) $$\Phi PSII=\frac{F_{m}^{\prime }-F_{s}}{{F_{m}}^{\prime }}$$

Here, $F_{m}^{\prime }$ represents the maximum fluorescence yield of the leaf under light conditions, while $F_{s}$ denotes the steady-state fluorescence yield. To ensure data representativeness, measurements were taken from 10 tea leaves per DSZ, with a mean value used for analysis.

During the experiment, a small portion of data was missing due to occasional instrument malfunctions. In the preprocessing step, all missing values were carefully removed before analysis. A total of 876 LAI values, 881 PRI values, and 827 ΦPSII values were collected for analysis.

#### 2.3.2. UAV Image Acquisition

In this study, a DJI Phantom 4 Pro and a DJI Phantom 4 Multispectral (DJI, Shenzhen, China) were used to capture visible-light and multispectral data, respectively. The experimental design achieved centimeter-level resolution, enabling leaf-level assessments. For visible-light UAV flight missions, the flight altitude was set at 30 m. If the terrain variation within a site exceeded 20 m, a multi-altitude flight strategy was applied to ensure both the quality and completeness of the orthomosaic images. For multispectral UAV flights, an altitude of 10 m above the tea canopy was maintained to match the spatial resolution of visible-light images within each DSZ. The image processing workflow included camera calibration, geographic coordinate correction, and radiometric correction (applied only to multispectral images), ultimately generating orthomosaic images. The final image resolution was approximately 0.8 cm/pixel.

### 2.4. Image Processing

#### 2.4.1. Canopy Part Segmentation

**Figure 4:**
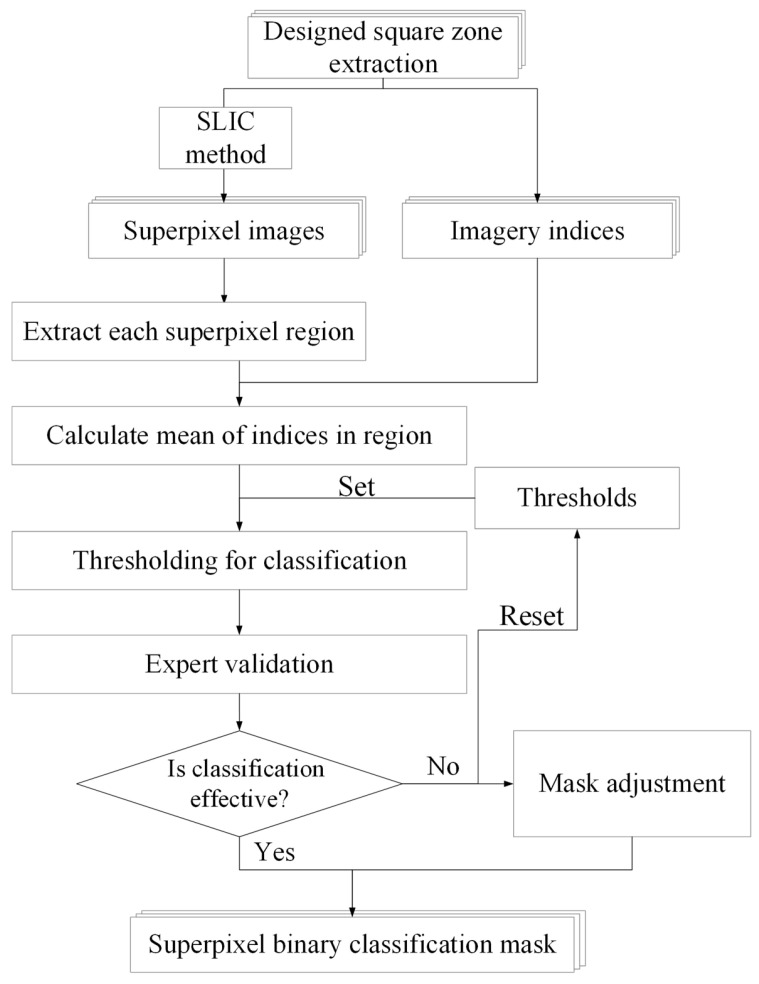
Workflow for tea canopy image classification.

High-resolution UAV imagery captures fine details but also includes non-target features such as soil, weeds, and irrigation structures [56]. The 21 tea plantations surveyed were managed by different tea farmers, resulting in diverse landscape characteristics across sites. To integrate UAV imagery from tea plantations across elevations and farming methods while reducing spectral influence from non-tea areas and improving future operational efficiency, this study developed an image-processing-based tea canopy classification method (Figure 4). This method first applies simple linear iterative clustering (SLIC) [57] for superpixel segmentation of DSZ images. Mean values of selected image indices are then calculated for each superpixel, and binarization thresholds are empirically adjusted. Experts manually review the results to ensure accuracy, segmenting images into target objects (tea plants) and non-target objects (soil, weeds, and artificial structures). This classification method offers four key advantages: (1) It eliminates the need for large labeled datasets, maintains controllable classification accuracy, and reduces data preparation time. (2) SLIC segmentation reduces shadow and gap interference within tea canopies, minimizing salt-and-pepper noise often caused by simple binarization. (3) It adapts to varying light conditions, enabling robust differentiation between vegetation and non-vegetation regions. (4) It ensures consistency across time-series data, allowing the same hyperparameters for classification within the same site across different time periods, thus supporting long-term monitoring and analysis.

The SLIC method is a K-means clustering technique that segments images into superpixels based on Lab color similarity and spatial pixel coordinates. The algorithm initially distributes cluster centers and iteratively updates only neighboring pixels, allowing cluster centers to converge in regions with similar color and spatial characteristics. Since SLIC operates locally, it is well-suited for high-spatial-resolution imagery, preserving important boundary information within the image. In this study, SLIC parameters were adjusted based on image characteristics, including the number of superpixels and the compactness factor. The number of superpixels was site-specific, while the compactness factor, which controls the shape of superpixels, was set to 20 for visible-light images and 0.05 for multispectral images. After segmentation, Otsu’s binarization method was applied using specific vegetation indices for classification. For visible-light images, the red–green–blue vegetation index (RGBVI) and the visible atmospherically resistant index (VARI) were used, while for multispectral images, the enhanced vegetation index (EVI) and the ratio vegetation index (RVI) were selected. Threshold values were fine-tuned for each site, with upper and lower limits set to exclude non-tea areas. When spectral reflectance made differentiation challenging, manual adjustments were incorporated to enhance segmentation accuracy.

#### 2.4.2. Calculation of Color Indices and Multispectral Indices

The index calculation for each DSZ image was based on the tea canopy segmentation results, meaning that only pixels identified as tea plants were used for index computation. The mean value of these indices within the tea plant regions was taken to represent the index value for each DSZ. For visible-light images, which consist of red, green, and blue bands, a total of 19 color indices (CIs) were computed. For multispectral images, which include five spectral bands—blue, green, red, red edge, and near-infrared (NIR)—a total of 50 multispectral indices (MIs) were derived. The names and formulas for the CIs and MIs are provided in Table A2.

### 2.5. Feature Ranking Methods

This study employed three feature ranking methods to analyze linearity, correlation and redundancy, and trend similarity, followed by ranking based on their results. These methods included: (1) Pearson correlation analysis (PCA), (2) Minimum Redundancy Maximum Relevance (MRMR) [58], and (3) Gray Relational Analysis (GRA). By applying the above-mentioned algorithms to assess the correlation between image indices and the target variables, the explanatory power of each index was determined, allowing for the assessment of the indices. Features were then sequentially added to the model based on their ranking to identify the optimal combination of independent variables for achieving the highest prediction accuracy.

#### 2.5.1. Pearson Correlation Analysis

Pearson correlation analysis (PCA) evaluates the linear relationship between two variables, with results expressed as the correlation coefficient (r). In this study, feature ranking was performed based on the absolute value of r. The formula for calculating r is as follows:

(3) $$r=\frac{\sum \left(x_{i}-\bar{x})(y_{i}-\bar{y}\right)}{\sqrt{\sum {\left(x_{i}-\bar{x}\right)}^{2}}\sqrt{\sum {\left(y_{i}-\bar{y}\right)}^{2}}}$$

where $x_{i}$ and $y_{i}$ represent the *i*th values of variables *x* and *y*, respectively, and $\bar{x}$ and $\bar{y}$ denote their respective mean values.

#### 2.5.2. Minimum Redundancy Maximum Relevance

Minimum redundancy maximum relevance (MRMR) simultaneously considers both the relevance of features to the target variable and the redundancy among features, aiming to select a feature subset with high relevance and low redundancy. MRMR is based on mutual information, which quantifies the dependency between two variables.

MRMR evaluates feature selection through two key components: (1) Max-Relevance and (2) Min-Redundancy. The core algorithm optimizes feature selection by maximizing the relevance of features to the target variable while minimizing redundancy among the selected features. The objective function is defined as follows:

(4) $$\underset{x_{j}\in X-S_{m-1}}{\max}\left[I\left(x_{j},\;y\right)-\frac{1}{m-1}\sum_{x_{i}\in S_{m-1}}I(x_{j},\;x_{i})\right]$$

where $I\left(x_{j},\;y\right)$ represents the mutual information value between the candidate feature $x_{j}$ and the target variable *y*, with higher values indicating greater relevance, and $\sum_{x_{i}\in S_{m-1}}I\left(x_{j},\;x_{i}\right)/\left(m-1\right)$ represents the mean mutual information between the candidate feature, (*x_j_*), and all features in the already-selected subset, $S_{m-1}$, with lower values indicating less redundancy.

#### 2.5.3. Gray Relational Analysis

Gray relational analysis (GRA) evaluates the trend similarity between features and target variables [23]. Since temporal variations may influence the relationships among indices, GRA can effectively identify key factors affecting a system [59]. GRA normalizes the dataset to eliminate numerical range differences and uses the gray relational coefficient (GRC) to quantify the similarity between each feature and the target variable, calculated as follows:

(5) $$\gamma_{0i}\left(k\right)=\frac{{∆}_{min}+\rho {∆}_{max}}{{∆}_{0i}(k)+\rho {∆}_{max}}$$

where $\gamma_{0i}\left(k\right)$ is the GRC of the *i*th feature at the *k*th data point, ${∆}_{min}$ and ${∆}_{max}$ are the minimum and maximum absolute differences among all sequences, ${∆}_{0i}(k)$ represents the absolute difference between the *i*th feature and the target variable at the *k*th point, and ρ is the distinguishing coefficient, which regulates the influence of the minimum and maximum differences on the correlation coefficient and is typically set to 0.5. The gray correlation degree (GCD) is computed to quantify the overall influence of a feature on the target variable and is calculated as follows:

(6) $$\gamma_{i}=\frac{1}{n}\sum_{k=1}^{n}\gamma_{0i}(k)$$

where $\gamma_{i}$ is the average GRC between the *i*th feature and the target variable, and n is the number of data points for the *i*th feature. Finally, features are ranked based on their GCD values, allowing for the identification of key features that most significantly influence the target variable [60].

### 2.6. Model Training and Evaluation Metrics

This study employed eight regression algorithms to explore the relationship between tea plant image indices and physiological parameters, including polynomial regression (PR), partial least squares regression (PLSR), lasso regression (LR), ridge regression (RR), decision tree regression (DTR), random forest regression (RFR), eXtreme gradient boosting (XGBoost), and the light gradient boosting machine (LightGBM). The hyperparameters adjusted for each model in this study are summarized in Table 1.

Polynomial regression (PR) is an extension of linear regression that incorporates higher-order terms of independent variables to fit nonlinear relationships, allowing the model to capture curved relationships among variables. Partial least squares regression (PLSR) integrates the strengths of principal component analysis, canonical correlation analysis, and multiple linear regression [61]. PLSR is particularly effective for handling multicollinearity among independent variables while reducing data dimensionality [62,63,64]. Lasso regression (LR) and ridge regression (RR) are regularized linear regression models but differ in their regularization approaches. Lasso regression applies an L1 regularization term in the loss function, forcing the coefficients of less important variables to shrink to zero [65,66], thereby performing automatic feature selection [67]. In contrast, ridge regression employs an L2 regularization term, which minimizes the loss function to handle multicollinear regression data, preventing overfitting when a large number of predictors are included [68]. The regularization strength for both models is controlled by the α parameter—higher values retain only the most influential variables, while lower values reduce regularization, increasing the risk of overfitting [69]. Decision tree regression (DTR) is a tree-structured model that simplifies complex decision-making through binary splits, progressively partitioning data into smaller subsets [70,71]. As decision trees are directly constructed from training samples, pruning is required to improve model generalization. Random forest regression (RFR) is an ensemble learning method based on decision trees [72] and is suitable for nonlinear and high-dimensional data. By constructing multiple randomized, decorrelated decision trees and averaging their results [73], RFR improves prediction performance while reducing overfitting using a bagging ensemble strategy [64,68,74]. eXtreme Gradient Boosting (XGBoost) is an enhanced gradient boosting algorithm [75] that divides the dataset into multiple subsets, trains base learners, and then aggregates their weighted predictions [74]. XGBoost runs efficiently and randomly selects feature indices to reduce overfitting risks. Light gradient boosting machine (LightGBM) is a high-efficiency ensemble learning algorithm built on the gradient boosting decision tree framework [76]. LightGBM employs a histogram-based decision tree algorithm, which accelerates node-splitting calculations by grouping features. Additionally, a leaf-wise growth strategy is used, splitting the leaf node with the highest gain, enabling the model to better capture nonlinear relationships in the data.

The dataset was trained and validated using three-fold cross-validation to reduce the risk of model overfitting. Model performance was evaluated using the coefficient of determination (R^2^), root mean square error (RMSE), and mean absolute error (MAE). The formula is as follows:

(7) $$R^{2}=1-\frac{\sum_{i=1}^{n}{\left(y_{i}^{m}-y_{i}^{p}\right)}^{2}}{\sum_{i=1}^{n}{\left(y_{i}^{m}-y_{i}^{a}\right)}^{2}}$$

(8) $$RMSE=\sqrt{\frac{1}{n}\sum_{i=1}^{n}{\left(y_{i}^{m}-y_{i}^{p}\right)}^{2}}$$

(9) $$MAE=\frac{1}{n}\sum_{i=1}^{n}\left|y_{i}^{m}-y_{i}^{p}\right|$$

where *i* represents the sample index, and *n* denotes the total number of samples, $y_{i}^{m}$ refers to the measured physiological parameter value of the *i*th sample, $y_{i}^{p}$ represents the predicted physiological parameter value of the *i*th sample, and $y_{i}^{a}$ denotes the average of the measured physiological parameter values in the dataset.

## 3. Results

### 3.1. Effects of Elevation Distribution and Farming Method Differences on Tea Plant Physiology

Table 2 presents the statistical data for three physiological parameters under two farming methods. The results indicate that the leaf area index (LAI) had a higher mean value under conventional farming (mean = 4.878) and exhibited a relatively stable data distribution (CV = 0.349). This stability may be associated with the management practices in conventional farming, which promote more uniform tea plant growth and reduce competition from weeds or other vegetation. In contrast, the mean quantum yield of PSII (ΦPSII) was higher under agroecological farming (mean = 0.4586), suggesting a potential advantage of this method in enhancing photosynthetic efficiency.

**Figure 5:**
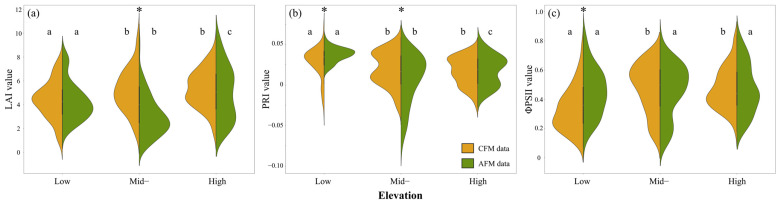
Violin plots and significance analysis of (**a**) LAI, (**b**) PRI, and (**c**) ΦPSII distributions. *: Significant difference between the two farming methods (*p* < 0.05); letters (a, b, c) denote significant differences among the three elevation levels within the same farming method (*p* < 0.05).

Figure 5 illustrates the distribution of the three physiological indices across low, mid-, and high elevations, comparing the measurement results between conventional and agroecological farming, along with the results of statistical significance testing. *T*-tests (* indicating significant differences between CFM and AFM at each elevation) showed that LAI differed significantly only at mid-elevation (*p* < 0.05), while PRI exhibited significant differences at both low and mid-elevations (*p* < 0.05). ΦPSII showed a significant difference only at low elevation (*p* < 0.05). To further analyze the variability across different elevations, a one-way analysis of variance (ANOVA) was performed. If the assumption of homogeneity of variance was not met, a Welch test was applied [77], with differences presented using the letter grouping method. The results indicated that under conventional farming, the three physiological parameters showed significant differences between low and mid-elevations, as well as between low and high elevations, while no significant differences were observed between mid- and high elevations. This result may be related to pruning practices in low-elevation plantations, where lower branches and older leaves are removed for management purposes. In contrast, higher-elevation plantations tend to have lower management intensity due to their larger area, allowing for the retention of larger canopy leaves, which could contribute to the overall higher LAI values. Under agroecological farming, both LAI and PRI exhibited significant differences across all three elevations, while ΦPSII did not show significant differences.

### 3.2. Feature Ranking of Image Indices

**Figure 6:**
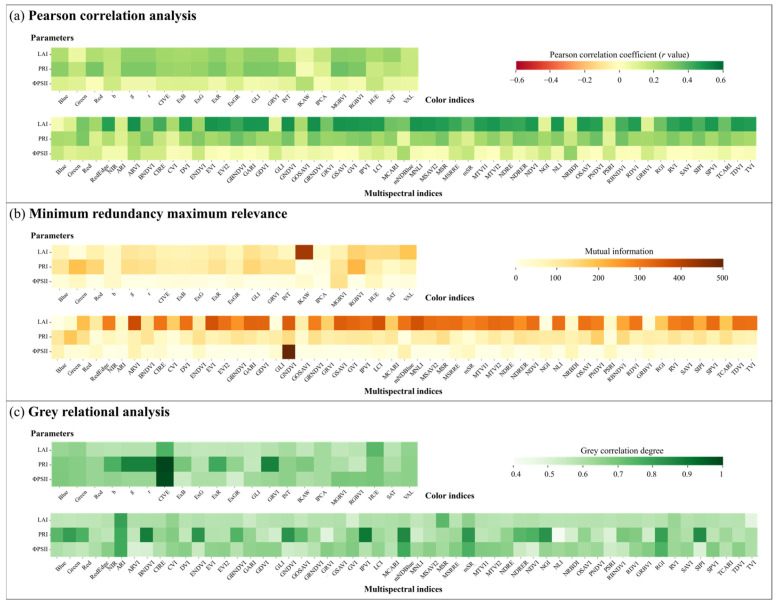
Heatmap of image indices importance based on (**a**) Pearson correlation, (**b**) minimum redundancy maximum relevance, and (**c**) gray relational analysis.

Figure 6 presents the heatmap of importance scores for three physiological parameters (LAI, PRI, and ΦPSII) based on three feature ranking methods, separately for two image datasets (CIs and MIs). The results show that compared to CIs, the importance scores of MIs are generally higher, indicating that multispectral images provide greater discriminative power and higher applicability in assessing plant physiological status.

### 3.3. Comparison of Regression Model Accuracy for Tea Plant Physiological Parameter Estimation

**Figure 7:**
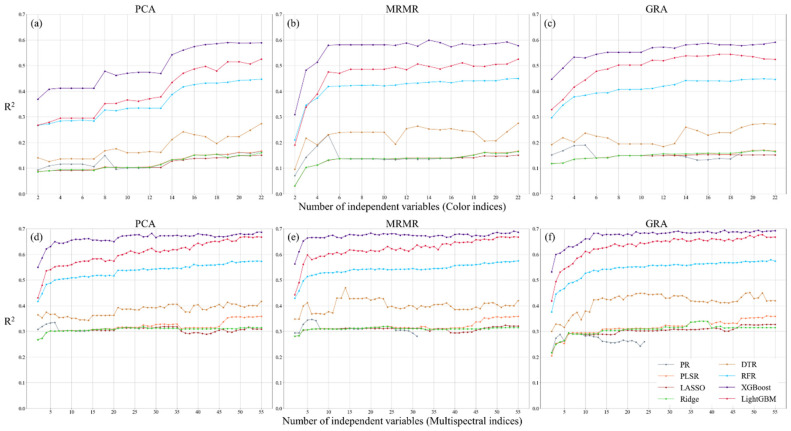
Regression accuracy of LAI using color (**a**–**c**) and multispectral indices (**d**–**f**) with three feature ranking methods.

**Figure 8:**
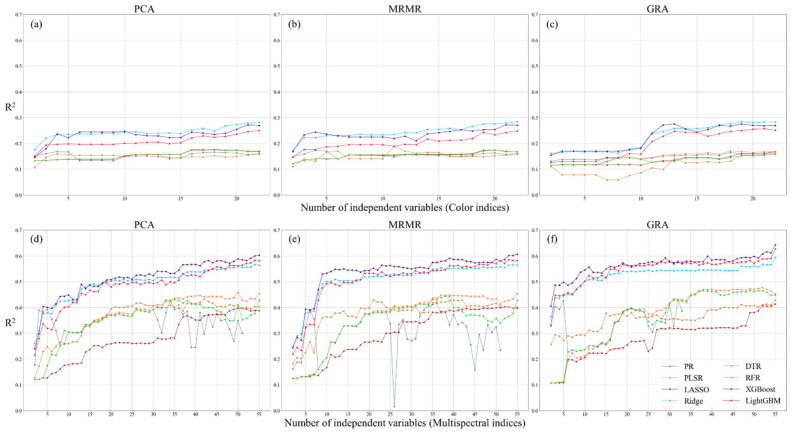
Regression accuracy of PRI using color (**a**–**c**) and multispectral indices (**d**–**f**) with three feature ranking methods.

**Figure 9:**
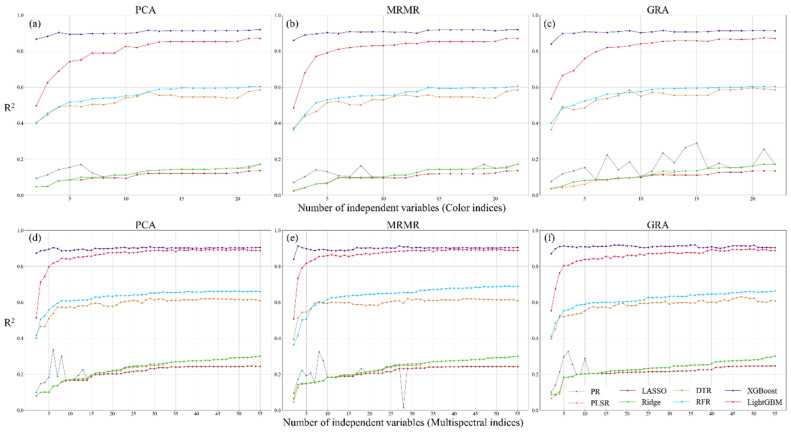
Regression accuracy of ΦPSII using color (**a**–**c**) and multispectral indices (**d**–**f**) with three feature ranking methods.

This study employed eight regression models to evaluate the predictive accuracy of CIs and MIs for estimating three physiological parameters (Figure 7, Figure 8 and Figure 9). Overall, model accuracy improved with an increase in the number of independent variables. However, the PR model exhibited unstable performance, with fluctuating accuracy values, indicating that simple nonlinear models have limitations in capturing the relationship between image indices and tea plant physiological parameters. In contrast, XGBoost, LightGBM, RFR, and DTR demonstrated slightly better accuracy than PLSR and the two regularized regression models (LR and RR), with XGBoost achieving the highest regression performance. The improvement in regression accuracy with XGBoost varied across different models. Compared to PR and PLSR, the accuracy gain ranged from 0 to 0.919 and 0.01 to 0.857, respectively. For regularized models (LR and RR), the improvement ranged from 0.01 to 0.849, while for decision tree-based models (DTR and RFR), the improvement was between 0.02 and 0.499. When compared to LightGBM, the accuracy gain was 0.006 to 0.375. Additionally, in the PR, PLSR, LR, and RR models, CIs struggled to effectively capture physiological parameter variations, while MIs often exhibited accuracy saturation. Although LightGBM and XGBoost performed comparably in most cases, XGBoost tended to capture physiological variations more efficiently, allowing it to achieve slightly better accuracy than LightGBM with fewer independent variables. This suggests that, given the same training and validation dataset, gradient boosting-based models demonstrate a higher capability in capturing the nonlinear relationships between tea plant physiological parameters and image indices. This can be attributed to the iterative optimization process of gradient boosting, which systematically reduces errors by compensating for uncaptured features and effectively accounting for interactions among variables.

This suggests that gradient boosting-based models are more effective at capturing the nonlinear relationships between tea plant physiological parameters and image indices. Regarding feature ranking methods, the differences in mean accuracy across the three methods were less than 0.099, indicating no significant differences. However, PCA-based ranking resulted in slightly lower prediction accuracy. The MRMR ranking method yielded more stable accuracy for CIs, while GRA-based ranking produced the best results for MIs. A comparison between the two types of image indices showed that using MIs generally resulted in higher prediction accuracy than using CIs. Specifically, this improvement was observed across all three physiological parameters, with LAI, PRI, and ΦPSII showing increases of approximately 15–115%, 98–179%, and 0–96%, respectively, when using MIs instead of CIs. These findings indicate that combining MIs with gradient boosting models provides greater suitability for capturing the nonlinear relationships of tea plant physiological parameters, while the choice of feature ranking method has a relatively minor impact on regression accuracy.

This study compares the performance of CIs and MIs in predicting three physiological parameters and evaluates regression accuracy using different feature ranking methods. The results indicate that the variation in model accuracy across different feature ranking methods was less than 0.04, suggesting that the chosen feature ranking approaches had no significant impact on regression accuracy in this study. For LAI regression performance, both CIs and MIs achieved an R^2^ exceeding 0.59, with RMSE ranging from 1.049 to 1.214 and MAE between 0.632 and 0.759. In PRI regression, the predictive power of CIs was weak (R^2^ = 0.28), whereas MIs improved R^2^ to 0.643, demonstrating the superior explanatory capability of multispectral data for PRI. At this stage, RMSE was 0.013, and MAE was 0.009, with MAE lower than the standard deviation of PRI under both farming methods (CFM: 0.0196, AFM: 0.0251), indicating that the prediction error was within an acceptable range. For ΦPSII prediction, both CIs and MIs achieved the highest predictive accuracy, with R^2^ exceeding 0.90, RMSE ranging from 0.048 to 0.052, and MAE between 0.013 and 0.024, indicating stable model performance. The optimal predictive results for the three physiological parameters using CIs and MIs indicate that the prediction accuracy of LAI and PRI improved by approximately 16.4% and 118.2%, respectively, with MIs, while RMSE decreased by 12.8% and 28.1%. For ΦPSII, the results indicate that the accuracy achieved using CIs and MIs was comparable. These findings suggest that MIs are more effective in capturing variations in tea plant physiological parameters in this study. Additionally, among all prediction results, only PRI prediction using CIs achieved the highest accuracy with the RFR model, while all other predictions achieved optimal accuracy using XGBoost. This highlights that gradient boosting regression models exhibit superior performance in predicting tea plant physiological parameters. The findings indicate that the models used in this study could explain 70–90% of the variance in LAI and ΦPSII data. While PRI predictions exhibited lower accuracy (approximately 65%), this level of precision remains within the acceptable range for crop physiological parameter modeling applications (Table 3).

This study further explored the relationship between the number of independent variables and regression accuracy, using the highest regression accuracy as a reference and setting 95% of this accuracy as the threshold to iteratively determine the number of independent variables required to meet this criterion. This approach reduces the number of independent variables while maintaining comparable accuracy. The results indicate that optimal accuracy is generally achieved when using more than half or even all variables, with GRA-based ranking performing best (see Table 4). In the MIs application, the highest accuracy for LAI and PRI was 0.716 and 0.643, respectively, with PRI showing a significant improvement of 0.359 compared to CIs. In contrast, the accuracy difference for ΦPSII between the two image datasets was minimal (0.001). When applying the 95% confidence interval as the accuracy threshold, the overall accuracy decreased slightly by approximately 0.014 to 0.046, but the number of required independent variables was significantly reduced. The number of independent variables for LAI and ΦPSII decreased by 32 and 19, respectively, while PRI required only 2 fewer variables. These findings suggest that image indices are more effective for predicting LAI and ΦPSII, whereas PRI may require additional information to accurately reflect its variations.

### 3.4. Effects of Elevation and Seasonal Conditions on Prediction Accuracy

**Figure 10:**
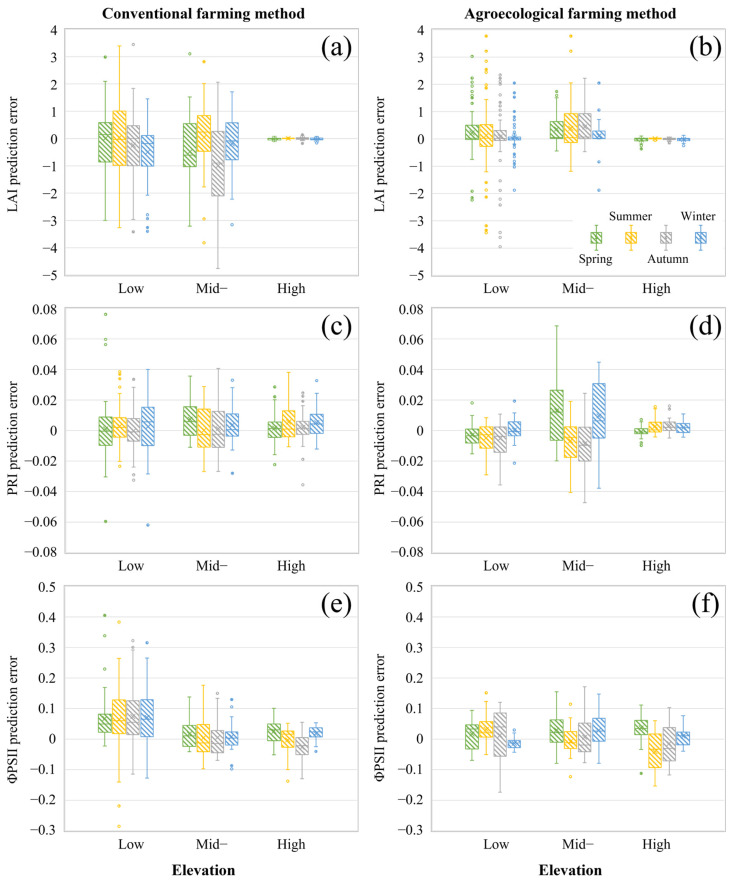
Boxplots of prediction errors for three physiological parameters across different elevations (low, mid, and high) and seasons (spring, summer, autumn, and winter) under conventional (**a**,**c**,**e**) and agroecological (**b**,**d**,**f**) farming methods.

Figure 10 illustrates the prediction errors of the best-performing regression model for estimating physiological parameters across different seasons. The results indicate that for LAI predictions, the conventional farming dataset exhibited a wider range of prediction errors (RMSE: 1.029–1.776), while the agroecological farming dataset contained more outliers. In terms of elevation, the high-elevation datasets from both farming methods showed the highest prediction accuracy across all seasons (RMSE: 0.021–0.107). At mid- and low-elevation plantations, the agroecological farming dataset exhibited smaller prediction errors (RMSE: 0.507–1.240), possibly due to lower intervention management strategies, which made plants more susceptible to field conditions and resulted in more extreme values. Additionally, predictions for conventional farming tended to be underestimated, which may be attributed to higher plant density and uniform growth, causing actual LAI values to be relatively high. Conversely, the overestimation observed in agroecological farming could be related to sparser plant distribution. Seasonal variations also influenced prediction accuracy, with higher prediction errors in summer and autumn, likely due to harvesting activities during the growing season, which made LAI estimation more complex.

For PRI predictions, the highest prediction errors occurred at mid-elevation plantations (RMSE: 0.013–0.026), whereas high-elevation plantations had the lowest prediction errors (RMSE: 0.003–0.015). Regarding farming methods, conventional farming exhibited relatively stable prediction errors across elevations and seasons (RMSE: 0.010–0.017). In contrast, for agroecological farming, low-elevation predictions were generally underestimated, while mid-elevation predictions showed higher errors in spring and winter (RMSE: 0.023–0.026) and lower errors in summer and autumn (RMSE: 0.015–0.018).

For ΦPSII predictions, the estimated values were slightly overestimated in most plantations. The agroecological farming dataset generally exhibited lower prediction errors (RMSE: 0.022–0.084), with autumn showing the highest errors among all seasons. In contrast, prediction errors for conventional farming (RMSE: 0.030–0.130) decreased with increasing elevation. These findings highlight the complex interactions between elevation, farming method, and seasonal effects on the prediction accuracy of tea plant physiological parameters.

## 4. Discussion

### 4.1. Differences in Tea Plant Physiological Parameters Across Elevations and Farming Methods

This study examines the physiological variations and differences in tea plants across elevations and two farming methods. Previous research has primarily focused on the impact of different farming methods on soil properties and yield differences [78,79], suggesting that these variations may result from a combination of geographical factors, crop types, and farming methods [80,81]. However, the physiological status of crops during their growth stages is a key determinant of yield and quality, yet it has received relatively less attention. Therefore, this study analyzes the physiological parameters of tea plants grown using CFMs and AFMs to explore the effects and differences.

The results indicate that at all elevations, LAI values were consistently higher in CFM than in AFM, with a significant difference observed only at mid-elevation. This phenomenon aligns with findings that tea yield under AFM conditions ranged from 20% to 80% of that under CFM conditions. Similarly, Schärer et al. [82] reported that winter wheat grown using organic farming had significantly lower LAI than that grown using conventional farming. Petcu et al. [83] further speculated that this discrepancy might be attributed to lower water and nutrient availability in organic farming systems, as organic fertilizers release nutrients more gradually, leading to relatively lower soil nitrogen content [84]. Olsen and Weiner [85] found that LAI is significantly correlated with nitrogen availability, and when nutrient supply is insufficient, leaf growth may be suppressed, directly impacting LAI performance. Although PRI and ΦPSII did not show significant differences between the two farming methods, values were generally higher in agroecological farming, suggesting that tea plants in AFMs may have advantages in physiological function and light energy utilization efficiency. Furthermore, tea plants grown using AFM exhibited greater physiological hardening effects in response to challenging environmental conditions than those grown using CFM. This suggests that under future extreme weather conditions, AFM helps maintain tea plant physiological health and stress resilience, offering greater long-term sustainability under global climate change conditions. Chen et al. [86] supported this perspective, showing that stomatal conductance in conventionally farmed tea plants was consistently lower than that in soil-cultured plants across all seasons, leading to reduced photosynthetic capacity and increased susceptibility to water stress. Over time, the sustained application of soil-based cultivation practices may improve tea plant resilience to climate change, particularly in ecosystems with irregular rainfall distribution. Overall, the results suggest that differences in tea plant physiological parameters between the two farming methods were relatively limited. However, although the yield under agroecological farming conditions is lower than that of conventional farming, it contains higher concentrations of antioxidant compounds, suggesting potential health benefits [84]. Additionally, the use of organic fertilizers significantly enhances tea quality and benefits soil fertility [87]. Furthermore, this study found that agroecological farming demonstrates advantages in environmental adaptability and light use efficiency, indicating that tea plants grown using agroecological farming are more resilient to environmental changes.

### 4.2. Effects of Feature Ranking Methods and Image Indices on Prediction Models

Feature selection is an effective approach for handling high-dimensional datasets, enabling the construction of a streamlined dataset that prioritizes variables with the greatest influence on the target variable [88]. The results indicate that the rankings produced by the three feature ranking methods varied, which is likely attributable to differences in algorithmic efficiency and performance characteristics [89]. This study also found that feature ranking methods had a limited impact on improving the accuracy of LR and RR models, a trend similarly observed by Shahsavari et al. [90]. This is primarily because regularization algorithms inherently balance feature importance through penalty terms, making the effect of pre-selection relatively insignificant for these models.

Furthermore, this study revealed that when using MIs to estimate physiological parameters, GRA-based feature ranking consistently yielded the highest regression accuracy. However, GRA has limitations when the relationship between independent and dependent variables is weak, making it less effective in distinguishing feature importance [91]. For instance, when CIs were used as independent variables, LAI and ΦPSII achieved the best performance using MRMR and Pearson correlation-based rankings, respectively. Overall, the impact of feature ranking methods on model accuracy was relatively minor in this study, whereas reducing the number of independent variables improved computational efficiency. Future research should consider the application when selecting appropriate feature selection methods, ensuring that the chosen approach effectively identifies key features and evaluates their influence on prediction accuracy.

### 4.3. Applicability Analysis of Regression Models

This study compared the performance of eight regression models to evaluate their applicability in predicting tea plant physiological parameters. The results indicate that the PR model exhibited lower accuracy and greater fluctuations, likely due to its sensitivity to multicollinearity and its limited ability to effectively model the relationship between image indices and crop physiological parameters [92]. In contrast, machine learning algorithms demonstrated superior performance in establishing relationships between image indices and crop physiological parameters, making them a more suitable choice for physiological parameter estimation [93]. When MIs were used for estimating physiological parameters, LAI prediction accuracy improved by approximately 0.095, with the largest improvement observed for PRI (0.359), while ΦPSII was less affected, highlighting the advantages of multispectral data in capturing crop physiological characteristics. Similarly, Zhang et al. [94] reported that multivariate regression models using multispectral data outperformed those based on visible-light data.

Our results also show that increasing the number of selected features generally improves model accuracy, but the magnitude of improvement is limited. To address this, we compared the number of features required for optimal accuracy versus a 5% reduction in accuracy. For LAI and ΦPSII, reducing accuracy by 5% resulted in a 60–90% reduction in the number of required features, providing advantages in feature computation and model processing time. Duro et al. [95] similarly found that removing 30–60% of irrelevant variables improved model interpretability while reducing accuracy by only 0.5%. Additionally, model performance metrics indicate the suitability of a method rather than its superiority. While an R^2^ value closer to 1 suggests higher accuracy, for high-variability systems, an R^2^ range of 0.7–0.9 is generally acceptable [96]. In wheat LAI prediction, Wu et al. [97] demonstrated that using a small set of UAV-derived features combined with multiple linear regression yielded efficient predictions (R^2^ = 0.679, RMSE = 1.231), while data fusion from multiple sensors further improved accuracy (R^2^ = 0.815, RMSE = 1.023). Wittstruck et al. [98] successfully predicted winter wheat LAI (R^2^ = 0.83, RMSE = 0.41) using UAV-derived visible-light data and plant height information. Similarly, Li et al. [99] applied visible UAV imagery, integrating CIs and texture features to estimate wheat LAI, achieving higher regression accuracy (R^2^ = 0.730, RMSE = 0.691) than models using individual features.

The results of this study show that while MIs significantly improved PRI prediction accuracy, their underlying physiological characteristics remain incompletely understood. Meanwhile, LAI and ΦPSII predictions reached acceptable accuracy levels. The best-performing model across all three physiological parameters was XGBoost, highlighting its capability to handle nonlinear relationships and complex feature interactions [100,101]. This demonstrates the effectiveness of XGBoost in achieving a strong generalization ability for predicting tea plant physiological parameters, providing a scientific foundation for future agricultural applications and model improvements.

## 5. Conclusions

This study utilized UAV-derived visible and multispectral imagery to estimate and analyze three physiological indices of tea plants (LAI, PRI, and ΦPSII). We evaluated color indices (CIs) and multispectral indices (MIs) and ranked their importance using Pearson correlation analysis (PCA), minimum redundancy maximum relevance (MRMR), and gray relational analysis (GRA) to develop predictive models for tea plant physiological parameters. Statistical analysis of tea plant physiological parameters revealed that while tea plants grown using agroecological farming methods (AFMs) did not show an advantage in external characteristics, this difference was indirectly reflected in yield variations between conventional farming methods (CFMs) and AFMs. However, physiological parameters related to light use efficiency and environmental adaptability suggest that tea plants grown using AFMs exhibit slightly higher resilience to environmental conditions, potentially offering greater long-term ecological benefits. The feature ranking results indicate that GRA achieved the best performance, while MIs exhibited a more robust explanatory capability for tea plant physiological parameters compared to CIs derived from visible-light imagery. Regarding regression model performance, XGBoost demonstrated superior predictive accuracy across all three physiological parameters. Based on the model configurations, the best-performing combinations for each physiological parameter were: (1) LAI: MI-GRA-XGBoost (R^2^ = 0.716, RMSE = 1.01, MAE = 0.683), (2) PRI: MI-GRA-XGBoost (R^2^ = 0.643, RMSE = 0.013, MAE = 0.009), and (3) ΦPSII: CI-PCA-XGBoost (R^2^ = 0.920, RMSE = 0.048, MAE = 0.013). These findings demonstrate the advantage of combining multispectral data with gradient boosting models for effectively capturing the complex physiological characteristics of tea plants.

This study framework focuses on developing a generalizable predictive model for tea plant physiological parameters to assess the feasibility of remote sensing-based monitoring methods for broader applications in tea plantations. The core objective is to evaluate the strategy of reducing the number of independent variables by allowing a slight trade-off in accuracy within an acceptable prediction error range while assessing the performance of various basic regression algorithms. Therefore, future research may refer to the feature selection approach used in this study to identify optimal features and further explore ensemble models or deep learning techniques to enhance predictive performance. The findings validate the potential of integrating multispectral data with feature ranking methods for predicting tea plant physiological parameters, providing a scientific reference for data-driven management and applications in precision agriculture.

## Figures and Tables

**Table 1:** Hyperparameters adjusted for each model.

Model	Hyperparameter
Polynomial Regression	Polynomial Terms: 2nd degree
Partial Least Squares Regression	n_components: from 2 to N based on the input features
Lasso Regression	Regularization strength (α): 0, 0.01, 0.1, 1, 10, 100
Ridge Regression	Regularization strength (α): 0, 0.01, 0.1, 1, 10, 100
Decision Tree Regression	max_depth: 4~100 min_samples_split: 5~50, increasing in increments of 5
Random Forest Regression	n_estimators: 50~150, increasing in increments of 25 max_depth: 3~50 min_samples_split: 5~50, increasing in increments of 5
eXtreme Gradient Boosting	max_depth: 3~25 learning_rate: 0.001, 0.005, 0.01, 0.05, 0.1 n_estimators: 50~150, increasing in increments of 25
Light Gradient Boosting Machine	num_leaves: 50~150, increasing in increments of 10 learning_rate: 0.001, 0.005, 0.01, 0.05, 0.1 n_estimators: 50~150, increasing in increments of 25

**Table 2:** Summary of descriptive statistics for LAI, PRI, and ΦPSII under two farming methods.

Parameter	Farming Method	Number	Min	Mean	Max	StDev	CV
LAI	CFM	499	0.500	4.878	10.370	1.704	0.349
	AFM	377	0.130	4.058	9.810	2.035	0.501
PRI	CFM	503	−0.0371	0.0228	0.0766	0.0196	0.860
	AFM	378	−0.0770	0.0201	0.0596	0.0251	1.249
ΦPSII	CFM	477	0.0729	0.4121	0.8277	0.1671	0.405
	AFM	350	0.0819	0.4586	0.8648	0.1717	0.374

**Table 3:** Accuracy of three physiological parameters under different image indices (CIs and MIs) and feature ranking methods.

Parameter	Index	Feature Ranking	R^2^	RMSE	MAE	Model
LAI	CI	PCA	0.59	1.214	0.759	XGBoost
		**MRMR**	**0.599**	**1.2**	**0.752**	
		GRA	0.591	1.212	0.751	
	MI	PCA	0.687	1.06	0.676	XGBoost
		MRMR	0.691	1.053	0.669	
		**GRA**	**0.694**	**1.049**	**0.632**	
PRI	CI	PCA	0.281	0.019	0.014	RFR
		**MRMR**	**0.284**	**0.019**	**0.014**	
		**GRA**	**0.284**	**0.019**	**0.014**	
	MI	PCA	0.603	0.014	0.009	XGBoost
		MRMR	0.607	0.014	0.009	
		**GRA**	**0.643**	**0.013**	**0.009**	
ΦPSII	CI	**PCA**	**0.92**	**0.048**	**0.013**	XGBoost
		MRMR	0.919	0.049	0.016	
		GRA	0.915	0.05	0.014	
	MI	PCA	0.909	0.052	0.021	XGBoost
		MRMR	0.913	0.05	0.024	
		**GRA**	**0.919**	**0.049**	**0.015**	

The bolded words in the table indicate the best results for this physiological parameter in CI/MI.

**Table 4:** Comparison of regression models, feature ranking methods, and accuracy metrics (best accuracy and 95% confidence interval) for tea plant physiological parameters.

Parameter	Indices	Feature Ranking	Accuracy ^1^	Model	# Variables ^1^ (Difference)
LAI	CI	MRMR	0.599/0.569	XGBoost	14/5 (9)
	**MI**	**GRA**	**0.716/0.680**		**43/11 (32)**
PRI	CI	**GRA**	0.284/0.270	RFR	19/17 (2)
	**MI**		**0.643/0.611**	XGBoost	**55/53 (2)**
ΦPSII	**CI**	**PCA**	**0.920/0.874**	XGBoost	**22/3 (19)**
	MI	GRA	0.919/0.873		36/3 (33)

^1^ Best accuracy/95% confidence interval lower bound. The bolded words in the table indicate the best results for this physiological parameter in CI/MI.

## Data Availability

Data are included in the article. Further inquiries can be made to the corresponding author upon request.

## Appendix A

**Table A1:** Basic information for each tea plantation.

Low elev.	Field	CLS001	CLS002		CLS003		CLS004			CLJ001		ALS001			ALS002			ALS003
	Area	3399	1142		1616		1110			6499		1651			2697			3065
	Slope	1.74	3.04		4.12		1.96			4.17		1.74			1.36			4.19
	Elev.	329	279		388		324			371		315			348			288
Mid-elev.	Field	CMC001		CMJ001		CMJ002			AMC001				AMJ001			AMJ002		
	Area	1131		1658		1911			1131				1658			1911		
	Slope	1.04		16.87		3.70			1.04				16.87			3.70		
	Elev.	565		826~833		924~926			565				826~833			924~926		
High elev.	Field	CHO001	CHO002		CHO003			AHO001			AHO002			AHO003			AHJ001	
	Area	28,749	5122		16,138			3412			10,744			4805			2082	
	Slope	42.35	36.83		24.35			18.19			35.65			20.63			27.25	
	Elev.	1510~1572	1406~1451		1381~1414			1523~1536			1476~1508			1452~1467			1471~1478	

Field naming: The first letter: “C” represents conventional farming tea plantations, while “A” represents agroecological farming tea plantations. The second letter: “L” means low elevation, “M” means mid-elevation, and “H” means high elevation. The third letter: “S” denotes Sijichun, “J” denotes Jinxuan, “C” denotes Chin-Shin-Dapan, and “O” denotes Chin-Shin-Oolong. Uniy: Area (m^2^); Slope (%); Elev. (m).

**Table A2:** Names and formulae of color indices (CIs) and multispectral indices (MIs).

Color Indices (CIs)	
Normalized Blue	$b=\frac{B}{R+G+B}$
Normalized Green	$g=\frac{G}{R+G+B}$
Normalized Red	$r=\frac{R}{R+G+B}$
Color Index of Vegetation	CIVE=0.441×r−0.811×g+0.3856×b+18.78745
Excess Blue Vegetation Index	ExB=1.4×b−g
Excess Green Vegetation Index	ExG=2×g−r−b
Excess Red Vegetation Index	ExR=1.4×r−g
Excess Green Minus Excess Red Index	ExGR=ExG−ExR
Green Leaf Index	
Green–Red Vegetation Index	$GLI=\frac{2\times G-R-B}{2\times G+R+B}$
Color Intensity Index	$INT=\frac{R+G+B}{3}$
Kawashima Index	$IKAW=\frac{R-B}{R+B}$
Principal Component Analysis Index	$IPCA=0.994\times \left|R-B\right|+0.961\times \left|G-B\right|+0.914\times \left|G-R\right|$
Modified Green–Red Vegetation Index	$MGRVI=\frac{G^{2}-R^{2}}{G^{2}+R^{2}}$
Red–Green–Blue Vegetation Index	$RGBVI=\frac{G^{2}-B\times R}{G^{2}+B\times R}$
Hue	$\theta ={cos}^{-1}\left\{\frac{\frac{1}{2}\left[\left(R-G\right)+\left(R-B\right)\right]}{\sqrt{{\left(R-G\right)}^{2}+\left(R-B\right)\left(G-B\right)}}\right\}$ $HUE=\left\{\begin{array}{c} \theta \\ 360{}^{\circ}-\theta \end{array}\begin{array}{c} if\\ if \end{array}\begin{array}{c} B\leq G\\ B>G \end{array}\right\}$
Saturation	$SAT=1-\frac{3}{R+G+B}min\left(R,G,B\right)$
Value	$VAL=\frac{1}{3}\left(R+G+B\right)$
Multispectral indices (MIs)	
Anthocyanin Reflectance Index	$ARI=\frac{\left(RE-G\right)}{\left(G\times RE\right)}$
Atmospherically Resistant Vegetation Index	$ARVI=\frac{\left[NIR-\left(R-2\times \left(B-R\right)\right)\right]}{\left[NIR+\left(R-2\times \left(B-R\right)\right)\right]}$
Blue Normalized Difference Vegetation Index	$BNDVI=\frac{NIR-B}{NIR+B}$
Chlorophyll Index Red Edge	$CIRE=\frac{NIR}{RE}-1$
Chlorophyll Vegetation Index	$CVI=\frac{\left(NIR\times R\right)}{\left(G^{2}\right)}$
Difference Vegetation Index	DVI=NIR−R
Enhanced Normalized Difference Vegetation Index	$ENDVI=\frac{\left[\left(NIR+G\right)-2\times B\right]}{\left[\left(NIR+G\right)+2\times B\right]}$
Enhanced Vegetation Index	$EVI=\frac{2.5\times \left(NIR-R\right)}{\left(NIR+6\times R-7.5\times B+1\right)}$
Enhanced Vegetation Index 2	$EVI2=\frac{2.5\times \left(NIR-R\right)}{\left(NIR+2.4\times R+1\right)}$
Green–Blue Normalized Difference Vegetation Index	$GBNDVI=\frac{NIR-(G+B)}{NIR+(G+B)}$
Green Atmospherically Resistant Index	$GARI=\frac{NIR}{G}-1$
Green Difference Vegetation Index	GDVI=NIR−G
Green Leaf Index	$GLI=\frac{2\times G-R-B}{2\times G+R+B}$
Green Normalized Difference Vegetation Index	$GNDVI=\frac{\left(NIR-G\right)}{\left(NIR+G\right)}$
Green Optimized Soil-Adjusted Vegetation Index	$GOSAVI=\frac{G-R}{G+R+0.16}$
Green–Red Normalized Difference Vegetation Index	$GRNDVI=\frac{NIR-(G+R)}{NIR+(G+R)}$
Green–Red Vegetation Index	$GRVI=\frac{\left(G-R\right)}{\left(G+R\right)}$
Green Soil-Adjusted Vegetation Index	$GSVAI=\frac{1.5\times (NIR-G)}{NIR+G+0.5}$
Green Vegetation Index	$GVI=\frac{NIR}{G}$
Infrared Percentage Vegetation Index	$IPVI=\frac{NIR}{NIR+R}$
Leaf Chlorophyll Index	$LCI=\frac{NIR-RE}{NIR+R}$
Modified Chlorophyll Absorption in Reflectance Index	$MCARI=\left(RE-R\right)-0.2\times (RE-G)\times \frac{RE}{R}$
Modified Normalized Difference Blue Index	$mNDBlue=\frac{\left(B-RE\right)}{\left(B+NIR\right)}$
Modified Nonlinear Vegetation Index	$MNLI\frac{1.5\times ({NIR}^{2}-R)}{\left({NIR}^{2}+R+0.5\right)}$
Modified Soil-Adjusted Vegetation Index 2	$MSAVI2=\frac{2\times NIR+1-\sqrt{{\left(2\times NIR+1\right)}^{2}-8\times (NIR-R)}}{2}$
Modified Simple Ratio	$MSR=\frac{\frac{NIR}{R}-1}{\sqrt{\frac{NIR}{R}}+1}$
Red Edge Modified Simple Ratio	$MSRRE=\frac{\frac{NIR}{RE}-1}{\sqrt{\frac{NIR}{RE}}-1}$
Modified Red Edge Simple Ratio	$mSR=\frac{NIR-B}{RE-B}$
Modified Triangular Vegetation Index 1	$MTVI1=1.2\times \left[1.2\times \left(NIR-G\right)-2.5\times (R-G)\right]$
Modified Triangular Vegetation Index	$MTVI2=\frac{1.5\times \left[1.2\times \left(NIR-G\right)-2.5\times (R-G)\right]}{\sqrt{{\left(2\times NIR+1\right)}^{2}-\left(6\times NIR-5\sqrt{R}\right)-0.5}}$
Normalized Difference Red Edge Index	$NDRE=\frac{NIR-RE}{NIR+RE}$
Normalized Difference Red Edge/Red	$NDRER=\frac{RE-R}{RE+R}$
Normalized Difference Vegetation Index	$NDVI=\frac{NIR-R}{NIR+R}$
Normalized Green Intensity	$NGI=\frac{G}{R+G+B}$
Nonlinear Vegetation Index	$NLI=\frac{{NIR}^{2}-R}{{NIR}^{2}+R}$
Normalized Red–Blue Difference Index	$NRBDI=\frac{G-B}{G+B}$
Optimized Soil-Adjusted Vegetation Index	$OSAVI=\frac{1.16\times (NIR-R)}{\left(NIR+R+0.16\right)}$
Pan Normalized Difference Vegetation Index	$PNDVI=\frac{NIR-(G+R+B)}{NIR+(G+R+B)}$
Plant Senescence Reflectance Index	$PSRI=\frac{R-G}{RE}$
Red–Blue Normalized Difference Vegetation Index	$RBNDVI=\frac{NIR-(R+B)}{NIR+(R+B)}$
Renormalized Difference Vegetation Index	$RDVI=\frac{NIR-R}{\sqrt{NIR+R}}$
Red–Green–Blue Vegetation Indices	$RGBVI=\frac{G^{2}-B\times R}{G^{2}+B\times R}$
Red–Green Index	$RGI=\frac{R}{G}$
Ratio Vegetation Index	$RVI=\frac{NIR}{R}$
Soil-Adjusted Vegetation Index	$SAVI=\frac{1.5\times (NIR-R)}{\left(NIR+R+0.5\right)}$
Structure Insensitive Pigment Index	$SIPI=\frac{\left(NIR-B\right)}{\left(NIR-R\right)}$
Spectral Polygon Vegetation Index	$SPVI=0.4\times (3.7\times \left(NIR-R\right)-1.2\times \left|G-R\right|)$
Transformed Chlorophyll Absorption in Reflectance Index	$TCARI=\frac{3\times \left[\left(RE-R\right)-0.2\times \left(RE-G\right)\times \left(RE/R\right)\right]}{\left[1.16\times (NIR-R)/NIR+R+0.16\right]}$
Transformed Difference Vegetation Index	$TDVI=\frac{1.5\times (NIR-R)}{\sqrt{{NIR}^{2}+R+0.5}}$
Triangular Vegetation Index	$TVI=60\times \left(NIR-G\right)-100\times (R-G)$
